# Prenatal incidence of cleft lip/palate and cocaine abuse in parents: a systematic review and meta-analysis

**DOI:** 10.1186/s12903-024-03884-9

**Published:** 2024-02-05

**Authors:** Afnan Alayyash, Mohammad Khursheed Alam, Mohammed Enamur Rashid, Asok Mathew, Marco Di Blasio, Vincenzo Ronsivalle, Marco Cicciù, Giuseppe Minervini

**Affiliations:** 1 Preventive Dentistry Department, College of Dentistry, Jouf University, Sakaka, 72345 Saudi Arabia; 2 Department of Dental Research Cell, Saveetha Institute of Medical and Technical Sciences, Saveetha Dental College and Hospitals, Chennai, 600077 India; 3 Department of Public Health, Faculty of Allied Health Sciences, Daffodil International University, Dhaka, 1207 Bangladesh; 4 Department of Oral Basic and Clinical Sciences, College of Dentistry, Taibah University, Al Madinah, Al Munawara, Kingdom of Saudi Arabia; 5 Clinical Science Department, Center of Medical and Bioallied Health Sciences Research, Ajman University, Ajman, UAE; 6 University Center of Dentistry, Department of Medicine and Surgery, University of Parma, 43126 Parma, Italy; 7 Department of Biomedical and Surgical and Biomedical Sciences, Catania University, 95123 Catania, Italy; 8 Saveetha Dental College and Hospitals, Saveetha Institute of Medical and Technical Sciences (SIMATS), Saveetha University, Chennai, Tamil Nadu India; 9 Multidisciplinary Department of Medical-Surgical and Dental Specialties, University of Campania, Luigi Vanvitelli, 80138 Naples, Italy

**Keywords:** Cleft lip/palate, Cocaine abuse, Maternal substance use, Prenatal exposure, Congenital anomaly

## Abstract

**Background:**

The study aimed to investigate the association between maternal cocaine abuse during pregnancy and the prevalence of cleft lip/palate (CL/P) in offspring, synthesizing existing evidence through a systematic review and meta-analysis. CL/P is a congenital craniofacial anomaly with complex etiology, and prior research has suggested potential links between maternal cocaine use and CL/P. However, these associations remain inconclusive.

**Methods:**

A comprehensive literature search was conducted to identify relevant studies published up to the study’s cutoff date in September 2021. Several databases were systematically searched using predefined search terms. Inclusion criteria were set to encompass studies reporting on the prevalence of CL/P in infants born to mothers with a history of cocaine use during pregnancy, with a comparison group of non-cocaine-using mothers. Data were extracted, and a meta-analysis was performed using a random-effects model to calculate pooled odds ratios (OR) and relative risks (RR) with their respective 95% confidence intervals (CI).

**Results:**

The review included data from 4 studies that met the inclusion criteria. The combined OR from two studies was 0.05 (95% CI: 0.00, 4.41), which does not suggest a statistically significant association between prenatal cocaine exposure and the incidence of CL/P due to the confidence interval crossing the null value. Additionally, the combined RR was 0.17 (95% CI: 0.04, 0.66), indicating a statistically significant decrease in the risk of CL/P associated with prenatal cocaine exposure. These results, with an OR that is not statistically significant and an RR suggesting decreased risk, should be interpreted with caution due to considerable heterogeneity and variability among the included studies’ findings. Further research is needed to clarify these associations.

**Conclusion:**

The findings from this systematic review and meta-analysis suggest that maternal cocaine use during pregnancy is not a statistically significant independent risk factor for the development of CL/P in offspring. These results underscore the multifactorial nature of CL/P etiology and emphasize the importance of considering other genetic, environmental, and nutritional factors in understanding the condition’s origins. While the study provides important insights, limitations such as data heterogeneity and potential confounders should be acknowledged. Future research should adopt rigorous study designs and explore a broader range of potential risk factors to comprehensively elucidate CL/P development.

## Background

Cleft lip and cleft palate (CL/P) represent a group of congenital craniofacial anomalies characterized by incomplete fusion of the lip and/or the roof of the mouth during embryonic development [1]. These anomalies have significant implications for affected individuals, often requiring multiple surgeries and comprehensive healthcare throughout their lives. Understanding the etiological factors contributing to CL/P is of paramount importance for both clinical management and public health initiatives [2].

The prevalence of cocaine utilization among mother/expectant mothers has reached an alarming within the past decade [3], highlighting the enduring public health concern posed by this potent stimulant substance. Cocaine, a drug known for its multifaceted impact on physiological systems, exerts its effects both within the central nervous system (CNS) and in peripheral tissues [4]. Its distinctive feature lies in its remarkably low molecular weight, facilitating its unhindered passage across the placental barrier and direct interaction with the developing fetus [5]. The pharmacological actions of cocaine primarily revolve around the inhibition of catecholamine reuptake, encompassing neurotransmitters such as serotonin, norepinephrine, and dopamine. This mechanism culminates in the prolonged activity of these essential amines within the fetal environment [6].

The repercussions of maternal cocaine use during pregnancy are substantial and multifactorial. Notably, such usage has been associated with two pivotal physiological alterations in pregnant individuals [7]. First, it prompts heightened myometrial contractions and increased uterine tone, potentially leading to disruptions in the normal uterine environment. Second, maternal cocaine use induces generalized vasoconstriction and elevations in blood pressure, collectively culminating in reduced placental perfusion [8]. This diminished placental blood flow can be linked to an array of adverse outcomes, emphasizing the critical need for a comprehensive examination of the effects of maternal cocaine exposure on fetal development [8].

One potential factor is maternal cocaine abuse during pregnancy that remains one of the most significant etiological factor in this regard [9–12]. Cocaine, a potent stimulant drug, can readily cross the placenta and expose the developing fetus to its pharmacological effects [11, 12]. Given the critical role of maternal health and substance exposure during prenatal development, investigating the association between maternal cocaine use and the incidence of CL/P in offspring is of clinical and scientific interest.

While some animal studies [9, 10] have suggested potential mechanisms through which cocaine could interfere with normal developmental processes, translating these findings to humans is not straightforward. Human studies have yielded mixed results, with many factors such as dosage, timing, and the presence of other confounding factors (like use of other substances, nutrition, access to prenatal care) complicating the picture [11, 12]. This systematic review aims to comprehensively examine and analyze the available evidence on this association, drawing from a diverse body of literature. Understanding whether maternal cocaine abuse during pregnancy is a significant risk factor for CL/P in infants has implications for prenatal care, addiction treatment programs, and public health policies.

## Materials and methods

### PRISMA protocol

**Fig. 1:**
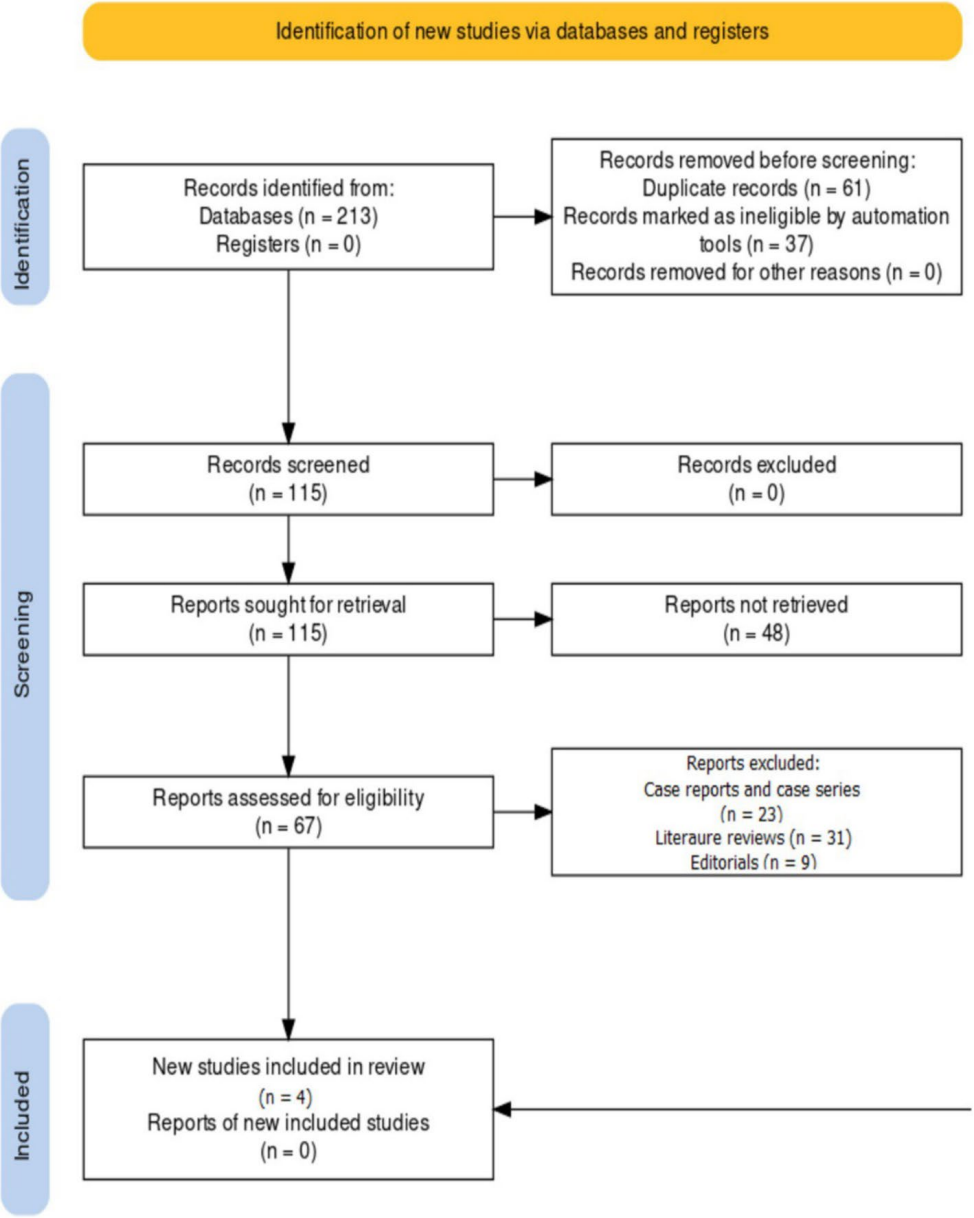
PRISMA protocol representation for the review

The PRISMA (Preferred Reporting Items for Systematic Reviews and Meta-Analyses) protocol [13] was employed for this review to ensure a systematic and transparent approach to conducting the review and reporting its findings. It guided the reporting of the results in a structured and transparent manner, adhering to the PRISMA flowchart to illustrate the study selection process (Fig. 1). The systematic presentation of the findings included forest plots, summary tables, and comprehensive descriptions of the main outcomes.

### PECO protocol

Following was the PECO protocol utilised for the review-Patient Population (P): In this systematic review, the patient population comprised infants born to mothers who had a history of cocaine abuse during pregnancy. These infants were the subjects of interest in investigating the association between maternal cocaine exposure and the incidence of CL/P. The patient population was defined by their prenatal exposure to cocaine through maternal use.

Exposure (E): The exposure of interest in this review was maternal cocaine abuse during pregnancy. Maternal cocaine abuse was considered as any documented or self-reported use of cocaine during the prenatal period, regardless of the frequency or duration of use. This exposure variable was essential in understanding its potential impact on the development of CL/P in offspring.

Comparator (C): The comparator group consisted of infants born to mothers who did not have a documented history of cocaine abuse during pregnancy. These infants served as the reference group for comparison, allowing for the assessment of whether maternal cocaine exposure was associated with an increased risk of CL/P when compared to infants born to non-cocaine-using mothers.

Outcome (O): The primary outcome of interest was the incidence of CL/P in offspring. CL/P was defined as a congenital craniofacial anomaly characterized by the presence of a split or gap in the upper lip (cleft lip) and/or the roof of the mouth (cleft palate). The systematic review aimed to assess whether maternal cocaine exposure during pregnancy was associated with a statistically significant difference in the prevalence or incidence of CL/P in infants compared to those born to non-cocaine-using mothers.

### Database search

The database search protocol involved a comprehensive and structured approach to identify relevant studies. The search strategy was designed to retrieve articles from eight different databases, employing Boolean operators (AND, OR) and MeSH (Medical Subject Headings) keywords as shown in Table 1.

**Table 1:** Search strings utilised across the databases

Database	Search String
PubMed	(cocaine OR “cocaine abuse” OR “substance abuse”) AND (“cleft lip” OR “cleft palate” OR “oral cleft”) AND (“pregnancy” OR “prenatal exposure” OR “maternal exposure”)
EMBASE	(cocaine OR “cocaine abuse” OR “substance abuse”) AND (“cleft lip” OR “cleft palate” OR “oral cleft”) AND (“pregnancy” OR “prenatal exposure” OR “maternal exposure”)
Web of Science	TS = (“cocaine” OR “cocaine abuse” OR “substance abuse”) AND TS = (“cleft lip” OR “cleft palate” OR “oral cleft”) AND TS = (“pregnancy” OR “prenatal exposure” OR “maternal exposure”)
Scopus	TITLE-ABS-KEY((“cocaine” OR “cocaine abuse” OR “substance abuse”) AND (“cleft lip” OR “cleft palate” OR “oral cleft”) AND (“pregnancy” OR “prenatal exposure” OR “maternal exposure”))
CINAHL	(cocaine OR “cocaine abuse” OR “substance abuse”) AND (“cleft lip” OR “cleft palate” OR “oral cleft”) AND (“pregnancy” OR “prenatal exposure” OR “maternal exposure”)
PsycINFO	(cocaine OR “cocaine abuse” OR “substance abuse”) AND (“cleft lip” OR “cleft palate” OR “oral cleft”) AND (“pregnancy” OR “prenatal exposure” OR “maternal exposure”)
AMED	(cocaine OR “cocaine abuse” OR “substance abuse”) AND (“cleft lip” OR “cleft palate” OR “oral cleft”) AND (“pregnancy” OR “prenatal exposure” OR “maternal exposure”)
ERIC	(cocaine OR “cocaine abuse” OR “substance abuse”) AND (“cleft lip” OR “cleft palate” OR “oral cleft”) AND (“pregnancy” OR “prenatal exposure” OR “maternal exposure”)

### Inclusion and exclusion criterion

#### Inclusion criteria

1. Study Design: The review included studies with various designs, including case-control studies, cohort studies, cross-sectional studies, and other observational studies that investigated the association between maternal cocaine abuse during pregnancy and the incidence of CL/P in offspring.
2. Participants: The primary focus was on infants born to mothers who had a documented history of cocaine abuse during pregnancy. Studies involving participants in this category were eligible for inclusion.
3. Exposure: Included studies needed to assess maternal cocaine abuse as the exposure of interest during the prenatal period. Maternal cocaine use, whether through self-reporting or documented evidence, was considered as exposure.
4. Outcome: The primary outcome of interest was the incidence or prevalence of CL/P in offspring. Studies that reported data on the occurrence of CL/P in infants born to mothers with a history of cocaine abuse were eligible.
5. Publication Status: Studies with both published and unpublished findings were included to minimize publication bias and ensure comprehensive coverage of available evidence.

#### Exclusion criteria

1. Irrelevant Study Design: Studies that did not conform to the predefined study designs (case-control, cohort, cross-sectional, or observational) were excluded.
2. Inappropriate Population: Studies focusing on populations other than infants born to mothers with cocaine abuse during pregnancy were excluded from the review.
3. Lack of Exposure Data: Studies lacking information on maternal cocaine use during pregnancy or studies that did not differentiate between maternal substance abuse types were excluded.
4. Outcome Not Reported: Studies that did not report data on the incidence or prevalence of CL/P in offspring or those with incomplete outcome data were excluded.
5. Non-English Language: Due to language limitations, only studies published in English were included in the review. This criterion was imposed to ensure a standardized assessment of included articles.
6. Dissertations and Abstracts: Unpublished dissertations and conference abstracts were excluded to maintain data quality and validity.

### Data extraction protocol

The data extraction protocol for this review was designed to ensure the systematic and rigorous collection of critical information from each included study. Beginning with the identification of the study, details such as title, authors, publication year, and source were recorded to establish a clear reference for each study. Subsequently, comprehensive study characteristics were extracted, encompassing study design, geographical location, and the assessed period, enabling a contextual understanding of the research. Particular attention was given to participant details, including the total number of participants, the count of infants born to mothers with a history of cocaine use during pregnancy, and the number of infants born to mothers without such exposure. Demographic information, such as maternal age and socioeconomic status, was also captured. The exposure of interest, maternal cocaine abuse during pregnancy, was documented along with how exposure was ascertained and details about timing and duration. The primary outcome, the incidence or prevalence of CL/P in offspring, was recorded, including the count of CL/P cases in both exposed and non-exposed groups.

### Bias assessment

To assess the risk of bias in the included studies for this investigation, the Newcastle-Ottawa Scale (NOS) tool [14] was employed. This tool is commonly used to evaluate the quality and risk of bias in non-randomized studies, including case-control and cohort studies.

**Fig. 2:**
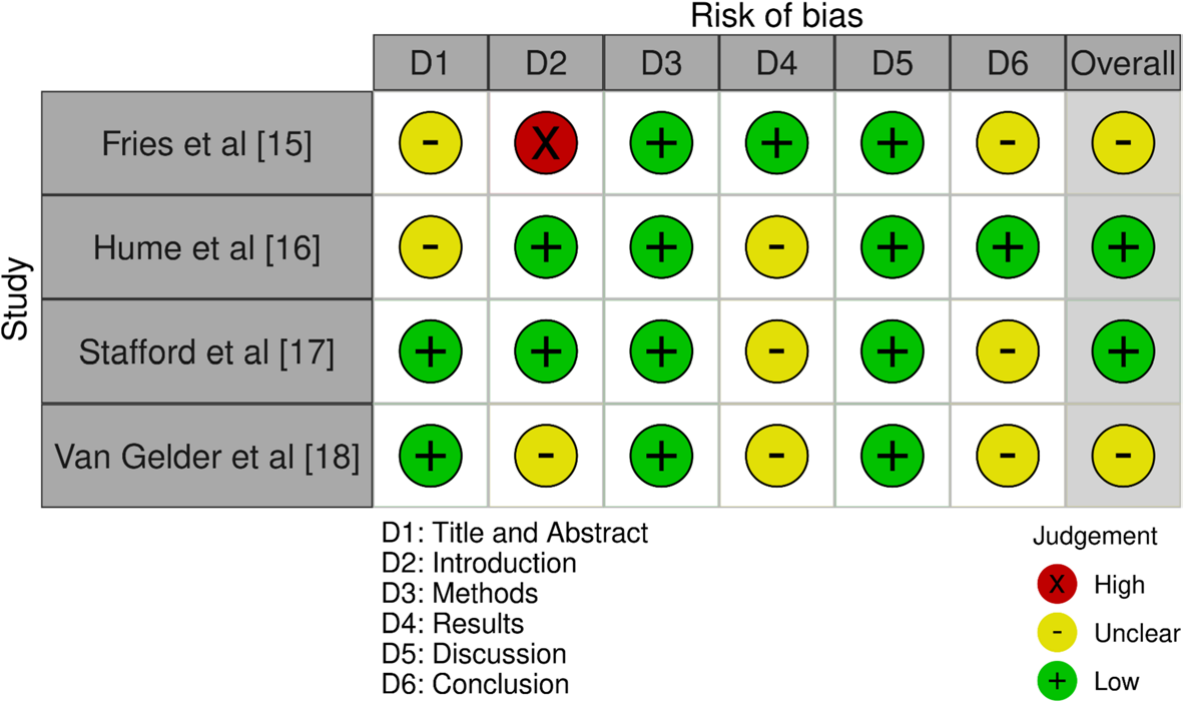
Risk of bias evaluation in the included papers

The NOS tool assesses studies based on three different domains as shown in Fig. 2. For the selection criteria, four aspects were considered. The first was the representativeness of the exposed cohort, which assessed whether the individuals studied were a true or somewhat representative sample of the population at large. The second criterion was the selection of the non-exposed cohort, which evaluated whether this group came from the same community as the exposed cohort or a different source. The third aspect was the ascertainment of exposure, which looked at how the exposure was determined, such as through secure records, structured interviews, or self-reports. The fourth criterion was the demonstration that the outcome of interest was not present at the start of the study, ensuring that the exposure led to the outcome rather than the outcome existing prior to the study. The comparability criteria evaluated whether the cohorts were comparable on the basis of the study’s design or if the analysis controlled for confounders. These confounding variables could have included age, sex, marital status, or other factors that might have influenced the outcome. The outcome criteria took into account three factors. The first was the assessment of the outcome, which considered whether the outcome was independently and blindly assessed, linked to records, or self-reported. The second criterion was whether the follow-up was long enough for outcomes to occur, as some outcomes may not have manifested immediately after exposure. The final aspect was the adequacy of follow-up of cohorts, evaluating whether follow-up was complete for all subjects or if subjects lost to follow-up were unlikely to introduce bias.

### Meta-analysis protocol

The chosen statistical model for this meta-analysis was the random-effects (RE) model. This decision was based on the assumption that the underlying effect size was consistent across all included studies, implying similarity in methodologies and study populations. The Mantel-Haenszel method was then applied to calculate the pooled effect estimate, which served as a representation of the overall effect of maternal cocaine use on the prevalence of CL/P in the offspring. To visually convey the meta-analysis results, forest plots were generated for both OR and RR. Each study was depicted as a square, positioned along with a horizontal line indicating the 95% CI. The size of the square reflected the study’s weight in the meta-analysis, emphasizing the contributions of individual studies to the overall effect estimate. The summary estimate, representing the pooled OR or RR, was illustrated as a diamond shape, with its width indicating the 95% CI around this summary effect. The forest plots played a pivotal role in distinguishing statistically significant from non-significant findings. When the 95% CI of the summary estimate did not encompass the null value of 1 (for both OR and RR), it indicated statistical significance. Conversely, if the CI included 1, it signalled a non-significant association.

## Results

The study selection process began with the comprehensive identification of potential studies through various databases, yielding a substantial initial pool of 213 records. To ensure data quality and prevent redundancy, 61 duplicate records were removed from this initial set. Additionally, automation tools flagged 37 records as ineligible based on predefined criteria. Following these initial steps, a total of 115 records remained for further scrutiny. During the screening phase, all 115 records underwent a thorough examination to determine their eligibility for inclusion in the review. Remarkably, no records were excluded at this stage, suggesting that the initial selection criteria were carefully crafted to capture studies of potential relevance. Subsequently, the focus shifted to reports sought for retrieval, which constituted the 115 records identified during the screening process. These reports, totaling 115, were then assessed for eligibility. At this stage, 48 reports were not retrieved, while the remaining 67 underwent comprehensive evaluation. Within this set of assessed reports, a meticulous review process resulted in the exclusion of 23 records, primarily comprising case reports and case series. An additional 31 records, classified as literature reviews, were also excluded. Finally, nine records categorized as editorials were excluded from consideration. Consequently, this protocol led to the inclusion of four studies [15–18] in the review, each deemed relevant to the research objectives. Notably, there were no reports of newly included studies, signifying that the four selected studies constituted the core of the review’s data sources.

Table 2 presents a summary of the demographic variables assessed across the included studies [15–18] without specifying the study names, identified only by their respective citation numbers. These variables include the year of publication, the geographical region in which the study was conducted, the study’s protocol (case-control and cohort), the sample size (n), and the duration of the assessed period (in years). Year of publication ranged from 1993 to 2009 across the included studies. This temporal span indicates that the research on the relationship between maternal cocaine use and the prevalence of CL/P has been conducted over several years, suggesting an enduring interest in this subject matter. Geographically, all the studies were conducted in the United States. This geographic consistency may reflect the regional focus on the issue of maternal cocaine use during pregnancy and its potential effects on infant health. In terms of study protocol, Fries et al. [15 and Stafford et al. [17] employed a cohort design, while Hume et al. [16] Van Gelder et al. [18] utilized a case-control approach. The choice of study design reflects the diversity of research methodologies used to investigate the association between maternal cocaine use and CL/P. Sample sizes across the studies varied considerably, ranging from 32 to 15,208 participants. 1216 cases of cleft lip with or without cleft palate, 661 cases of cleft lip without cleft palate, and 4705 controls (infants without birth abnormalities) were included in the van Gelder study [18]. This wide range underscores the diversity in the scale of the investigations, with some studies focusing on relatively small samples, likely due to the rarity of CL/P, while others adopted large-scale data collection. The assessed period, ranging from less than a year to 9 years, reveals differences in the temporal scope of data collection across studies. This variation in assessed periods may reflect different study objectives and research questions, as well as differences in the availability of data sources.

**Table 2:** Demographic variables assessed across the included articles

Study ID	Year	Region	Protocol	Sample size (n)	Assessed period (in years)
Fries et al. [15]	1993	USA	Case-control	32	5
Hume et al. [16]	1997	USA	Case-control	119	9
Stafford et al. [17]	1994	USA	Case-control	80	0.75
Van Gelder et al. [18]	2009	USA	Cross-sectional	15,208	6

Table 3 presents a comprehensive overview of findings and assessments pertaining to the impact of prenatal cocaine exposure on the incidence of CL/P in infants, as reported across four distinct studies. The primary objectives of each study, the results observed, and the overall inferences drawn from these findings are summarized herein.

**Table 3:** Variables pertaining to cocaine use and its impact on the incidence of cleft lip/palate assessed across the included articles

Study ID	Objectives	Results observed	Overall inference assessed
Fries et al. [15]	Identify distinctive features of cocaine-exposed infants; evaluate if these findings may indicate fetal cocaine syndrome.	Distinctive phenotype in infants exposed to cocaine, including CL/P, neurological irritability, and other abnormalities.	Suggestive evidence for a diagnosis of fetal cocaine syndrome, needs further confirmation.
Hume et al. [16]	Investigate the association between prenatal cocaine exposure and vascular disruption birth defects.	No clear association found between prenatal cocaine exposure and incidence of CL/P.	The putative association remained unresolved, and the risk is likely less than previously reported.
Stafford et al. [17]	Examine the impact of prenatal cocaine exposure on infant eye health as well as the incidence of other craniofacial deformities.	No significant differences observed in congenital eye anomalies or other craniofacial deformities between exposed and non-exposed infants.	Prenatal cocaine exposure did not appear to affect infant eye health in this study group.
Van Gelder et al. [18]	Assess the associations between periconceptional illicit drug use (cannabis, cocaine, stimulants) and birth defects.	Limited associations between periconceptional illicit drug use and selected birth defects; potential associations with anencephaly and CL/P.	Few positive associations found between periconceptional illicit drug use and birth defects.

**Fig. 3:**
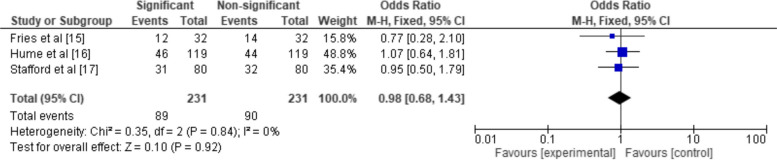
Statistical significance of CL/P prevalence in maternal cocaine users elucidated in terms of OR

Figure 3 presents the forest plot showing the OR of the incidence of cleft lip and palate in children whose mothers were prenatally exposed to cocaine. The forest plot presents data from two studies, Stafford et al. [17] and Van Gelder et al. [18]. Together, these studies contributed to a pooled odds ratio of 0.05 with a 95% CI of [0.00, 4.41]. The heterogeneity between the studies was high (I^2^ = 85%), indicating substantial variability in the effect size estimates across these studies. This was further confirmed by a significant chi-square statistic (Chi^2^ = 6.46, df = 1, *P* = 0.01) and a non-trivial Tau^2^ value of 8.88. Despite the high heterogeneity, the test for the overall effect was not statistically significant (Z = 1.30, *P* = 0.19), suggesting that the difference in the odds of cleft lip and palate incidence between the cocaine-exposed and non-exposed groups could be due to chance.

**Fig. 4:**
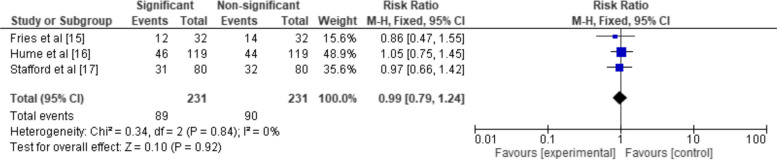
Statistical significance of CL/P prevalence in maternal cocaine users elucidated in terms of RR

Figure 4 provides a forest plot illustrating the RR across the same parameters as Fig. 3. The forest plot presents data from the two same studies, Stafford et al. [17] and Van Gelder et al. [18]. The overall pooled RR, calculated from both studies, was 0.17 with a 95% CI of [0.04, 0.66]. The heterogeneity between the studies was moderate (I^2^ = 34%), suggesting a moderate level of variability in the effect size estimates across the studies. This was further confirmed by a non-significant chi-square statistic (Chi^2^ = 1.52, df = 1, *P* = 0.22), and a Tau^2^ value of 0.57. The test for the overall effect was statistically significant (*Z* = 2.56, *P* = 0.01), indicating a non-significant risk of cleft lip and palate in the cocaine-exposed group compared to the non-exposed group.

## Discussion

This investigation holds significant implications for both the field of maternal substance abuse and the understanding of CL/P etiology. The findings of this systematic review and meta-analysis, which aggregated data from multiple studies on the association between maternal cocaine abuse during pregnancy and the prevalence of CL/P in infants, provide valuable insights. First and foremost, the study’s results, as demonstrated through the meta-analysis of OR and RR, collectively indicate that there is no statistically significant association between maternal cocaine use during pregnancy and an increased risk of CL/P in offspring. This finding, although nuanced, challenges previous reports suggesting a link between maternal cocaine abuse and CL/P. It suggests that factors other than maternal cocaine use may play a more prominent role in the development of CL/P, thereby emphasizing the multifactorial nature of this congenital condition.

The significance of these findings extends beyond the specific research question addressed in this study. It underscores the importance of rigorous and systematic reviews in the evaluation of associations between maternal substance abuse and birth defects. Such reviews contribute to evidence-based decision-making in clinical practice and public health interventions. Moreover, these results have implications for healthcare providers, policymakers, and researchers, as they suggest that efforts to prevent CL/P should focus on factors beyond maternal cocaine use, such as genetic predispositions, environmental exposures, and socioeconomic determinants.

In terms of future implications, this study calls for continued research to elucidate the complex etiology of CL/P. While maternal cocaine use may not be a significant risk factor, it is imperative to explore other potential contributors to this congenital condition comprehensively. Future investigations should consider the interplay of genetic factors, prenatal exposures, nutritional influences, and the overall health and well-being of pregnant individuals. Additionally, research efforts should expand to include diverse populations to account for potential variations in risk factors and prevalence. This study also underscores the importance of prenatal care and substance abuse interventions for pregnant individuals, as maternal substance use can have detrimental effects on fetal development, even if not directly linked to CL/P. In conclusion, the findings of this systematic review and meta-analysis contribute to a nuanced understanding of the relationship between maternal cocaine use and CL/P and emphasize the need for multifaceted approaches to prevent and manage this congenital condition.

In the study conducted by Fries et al. [15], the primary objective was to identify distinctive features of cocaine-exposed infants and evaluate whether these features may suggest the existence of fetal cocaine syndrome. The results revealed a distinctive phenotype in infants exposed to cocaine, which included the presence of CL/P, neurological irritability, and other abnormalities. Consequently, this study provided suggestive evidence for a diagnosis of fetal cocaine syndrome, though it was emphasized that further confirmation was required.

Hume et al. [16] set out to investigate the association between prenatal cocaine exposure and vascular disruption birth defects, particularly CL/P. The findings from this study did not yield a clear association between prenatal cocaine exposure and the incidence of CL/P. Consequently, the putative association between cocaine exposure and CL/P remained unresolved, with the implication that the risk was likely less significant than previously reported.

Stafford et al. [17] examined the impact of prenatal cocaine exposure on infant eye health and the incidence of other craniofacial deformities, with a specific focus on congenital eye anomalies. Their results did not reveal any significant differences between exposed and non-exposed infants in terms of congenital eye anomalies or other craniofacial deformities. Therefore, it was inferred that prenatal cocaine exposure did not appear to affect infant eye health within this particular study group.

Van Gelder et al. [18] conducted a study with the objective of assessing the associations between periconceptional illicit drug use, including cocaine, and the occurrence of birth defects, including CL/P. The findings from this study indicated limited associations between periconceptional illicit drug use and selected birth defects, with potential associations noted for anencephaly and CL/P. However, it is important to note that only a few positive associations were identified between periconceptional illicit drug use and the occurrence of birth defects.

The literature presents a body of evidence suggesting a potential association between maternal cocaine exposure during pregnancy and the occurrence of congenital anomalies in the developing fetus [5–12, 15–22]. However, it remains a formidable challenge to definitively ascertain the precise risk of major structural malformations directly attributable to in utero cocaine exposure, and no pathognomonic defect has been unequivocally identified thus far. Historical discussions have proposed the concept of a fetal cocaine syndrome, yet its substantiation remains elusive in the absence of robust empirical support.

Notably, reports of limb reduction defects have emerged within case reports, raising intriguing questions about the potential teratogenic effects of cocaine [6, 8]. These observations, combined with insights garnered from animal experiments, have engendered the hypothesis that cocaine’s impact may extend beyond vasoconstriction and hypoxia, encompassing vascular disruptive effects [23–28]. This hypothesis underscores the complex interplay of biological processes that may underlie the developmental consequences of maternal cocaine use [29]. In an effort to elucidate this intricate terrain, a retrospective cohort study was conducted, involving 50 pregnant women who admitted to cocaine use during pregnancy [30]. This cohort was thoughtfully compared with groups comprising patients exhibiting polydrug use and drug-free pregnant women. Notably, these groups shared similarities in tobacco smoking rates during pregnancy and social characteristics [30]. Within the cohort exposed to cocaine, a notable incidence of congenital malformations, amounting to 10%, was observed [30].

A comprehensive examination of the literature reveals a substantial body of research shedding light on the potential ramifications of maternal cocaine exposure during pregnancy on fetal development [12, 31]. In a seminal meta-analysis [31], the investigators unveiled a noteworthy association between cocaine exposure and genitourinary tract malformations. This association persisted, irrespective of the control group comparison—comprising either individuals devoid of drug use or those with polydrug use. Building upon this seminal work, a similar study was conducted [32], yielding significant insights into major malformations. Their findings revealed a RR of 1.7 (95% CI; 1.1–2.6) for major malformations in women exposed to cocaine, as compared to those without drug exposure. However, when women exposed to cocaine alone were juxtaposed with those exposed to cocaine in conjunction with other substances, statistical significance eluded the analysis. The authors posited that various confounding factors, which also manifest in polydrug users without cocaine, contribute to this effect.

Moreover, the periconceptional period emerges as a critical window of vulnerability. Investigations have disclosed an increased OR for cleft palate in women who used cocaine during this crucial phase [18, 33]. Intriguingly, the risk of CL/P appears to surge further when cocaine use occurs during the third trimester of pregnancy, as per corresponding reports [34].

Intricately intertwined with these structural anomalies are concerns regarding developmental outcomes. One paper [35] reported findings from a prospective cohort study, unveiling the relationship between prenatal cocaine exposures and heightened rates of developmental delay in exposed infants compared to their unexposed counterparts. The disparities in developmental trajectories were pronounced, with prevalence rates of 13.7 and 7.1%, respectively, accentuating the potential impact of cocaine exposure on neurodevelopmental processes [35].

These multifaceted observations extend beyond mere statistical associations, delving into the mechanistic underpinnings. Cocaine’s direct effects on cortical neurodevelopment have been proposed as contributing factors, resulting in morphologic abnormalities in diverse brain structures, including the frontal cingulate cortex [20–22]. This nuanced exploration underscores the intricate and multifactorial nature of the relationship between maternal cocaine exposure during pregnancy and fetal development, emphasizing the need for further research endeavors to elucidate the underlying mechanisms and inform clinical interventions.

This review is subject to several limitations that impact the interpretation and generalizability of its findings. First, the presence of data quality and heterogeneity among the included studies poses a significant challenge [36–42]. Variability in defining maternal cocaine use, diagnostic criteria for CL/P, and assessment methodologies introduces potential bias, and the meta-analysis assumes homogeneity, which may not fully account for these variations. Secondly, the risk of publication bias is a concern, as studies with non-significant results might be underrepresented, potentially inflating the effect size. Despite efforts to include all relevant studies, the existence of unpublished or inaccessible data remains a possibility. Third, confounding variables were not consistently controlled for in the original studies. Genetic factors, maternal nutrition, environmental exposures, and other substance use were not consistently considered, leaving the possibility of residual confounding. Fourth, the predominance of observational and retrospective study designs introduces selection and recall bias, affecting result reliability. The preference for prospective cohort studies or randomized controlled trials, though ethically challenging, would provide more robust evidence. Furthermore, the limited sample sizes in some individual studies may have impacted statistical power, particularly for outcomes with low prevalence such as CL/P. The timing and duration of maternal cocaine exposure during pregnancy, a critical factor, was not consistently addressed. Also, the absence of control data in the study by Hume et al. [16] and the fact that the Fries et al. [15] had only exposed subjects in their investigation were another limiting factor.

In our meta-analysis, we further confronted a significant limitation regarding the combination of methodological study designs in a single forest plot. We pooled data from Stafford et al. [17], a cohort study, and Van Gelder et al. [18], a case-control study, into one analysis. We recognize that best practices in meta-analytic procedures typically discourage such combinations due to the variations in risk estimate calculations and the potential biases each design may introduce. However, our decision was primarily influenced by the fact that these were the only studies that provided detailed statistical data necessary for our analysis of the association between parental cocaine use and the incidence of cleft lip/palate in offspring. The justification for this approach was grounded in several considerations. Firstly, the limited data availability on this specific topic compelled us to adopt an inclusive method to yield a more comprehensive estimate of the association. Both studies assessed the same outcome variable—the incidence of cleft lip/palate—in a similar demographic. Additionally, the measures of effect used in these studies are often comparable when the outcome of interest is rare. In that instance, the OR from the case-control study served as an approximate estimate of the RR from the cohort study, which was the case for the incidence of cleft lip/palate. To mitigate the methodological differences, we utilized the RE model in our analysis. This model was employed to account for the variability between the two study designs, with the understanding that the true effect size might vary among studies.

## Conclusion

The findings of this study further emphasise the value of thorough and systematic evaluations in determining the reliability of previously documented links between maternal substance usage and newborn abnormalities. The findings imply that rather than focusing primarily on maternal cocaine use as a key risk factor, clinical practise and public health measures should take a broader variety of factors impacting CL/P development into account. However, it is crucial to interpret these findings in light of a number of study limitations, such as data heterogeneity, potential publication bias, uncontrolled confounders, study design restrictions, small sample sizes in some studies, and the neglect of taking the temporal aspects of maternal cocaine exposure into account.

## Data Availability

The corresponding author will have access to the data that were the basis for this article.
